# Trends, Outcomes, and Predictors of Sepsis and Severe Sepsis in Patients with Left Ventricular Assist Devices

**DOI:** 10.7759/cureus.7523

**Published:** 2020-04-03

**Authors:** Karthik Gonuguntla, Shivaraj Patil, Naga Vaishnavi Gadela, Richard Cowden, Chaitanya Rojulpote, Satya Durugu, Pranav Karambelkar, Abhijit Bhattaru, Austin J Borja, Vincent Zhang

**Affiliations:** 1 Internal Medicine, UConn Health, Farmington, USA; 2 Internal Medicine, University of Connecticut, Farmington, USA; 3 Psychology, University of the Free State, Bloemfontein, ZAF; 4 Internal Medicine, The Wright Center for Graduate Medical Education, Scranton, USA; 5 Nuclear Cardiology and Cardiovascular Molecular Imaging, University of Pennsylvania, Philadelphia, USA; 6 Infectious Disease, University of Louisville, Louisville, USA; 7 Radiology, Hospital of the University of Pennsylvania, Philadelphia, USA; 8 Radiology, Perelman School of Medicine at the University of Pennsylvania, Philadelphia, USA

**Keywords:** end-stage heart failure, nis, sepsis, severe sepsis, complications, in-hospital mortality

## Abstract

Left ventricular assist device (LVAD) is used in end-stage heart failure that is refractory to medical treatment. However, there is a paucity of data looking at the rates of sepsis and severe sepsis (SSS). Therefore, this study was conceived with the purpose of analyzing the SSS burden and outcomes associated with LVAD implantation. The national inpatient sample database was queried from 2010 to 2014 using ICD-9 procedure code for LVAD use among patients 18 years or older and 2359 patients were identified. During the five-year study period, the average incidence of SSS was 11.8% and it was noted that cases with SSS were associated with an increased likelihood of mortality, greater length of hospital stay (LOS), and higher hospital-related charges (p < .001) compared to controls. Controlling for age, sex, and LOS, hierarchical multivariate logistic regression revealed that significant predictors of SSS were acute kidney injury [Adjusted odd’s ratio (AOR) = 2.75, 95% CI = 1.87, 4.14)], mechanical ventilation (AOR = 2.34, 95% CI = 1.70, 3.23), venous thromboembolism (AOR = 1.76, 95% CI = 1.12, 2.75), gastrointestinal bleed (AOR = 1.77, 95% CI = 1.12, 2.76), chronic obstructive pulmonary disease (COPD) (AOR = 0.55, 95% CI = 0.40, 0.77), acute myocardial infarction (AOR = 0.54, 95% CI = 0.36, 0.80) and mild liver disease (AOR = 2.18, 95% CI = 1.55, 3.06). The rate of incidence of sepsis has remained constant and is often associated with a worse clinical outcome. This provides a basis to identify high-risk groups and helps argue for earlier detection of such patients and better patient selection so as to reduce infectious complications.

## Introduction

A ventricular assist device is an electromechanical device for assisting cardiac circulation in end-stage heart failure that is refractory to medical treatment. It is used either to partially or completely replace the function of a failing heart. The continued evolution of left ventricular assist devices (LVADs) has led to major improvements in survival and quality of life. However, the risks of complications such as bleeding, infections, and immunosuppression persist even with newer devices. Patients with end-stage heart failure often have cardiac cachexia due to decreased nutritional intake, specific metabolic alterations which may predispose these patients to infectious complications [1]. Newer continuous-flow left ventricular assist devices have replaced the first-generation pulsatile flow devices and the rate of infections has decreased as much as 50% [2]. However, since left ventricular assist devices involve the use of a foreign body, the incidence of infectious complications and sepsis remains considerable. Infection is a common cause of morbidity and is the second most common cause of death in patients who survive the initial six months on continuous-flow - LVAD support [3]. Recent studies have shown an increase in the use of LVADs with improved survival rates but there is a paucity of data looking at the rates of sepsis and severe sepsis (SSS). Hence, the purpose of the study was to analyze the SSS burden and outcomes associated with LVAD implantation.

## Materials and methods

Data source

The data was obtained for the years 2010 to 2014 from the National Inpatient Sample (NIS) database. It is part of the Healthcare Cost and Utilization Project (HCUP) and sponsored by the Agency for Healthcare Research & Quality. The database was created by the agency for healthcare research and quality and contains data on 5 to 8 million hospital stays from approximately 1,000 hospitals. With the established weights in NIS, this sample could be weighted to represent the standardized U.S. population and contains approximately more than 35 million hospitalizations each year. It was designed to include data from a 20% sample of discharges from all participating hospitals and covers >95% of the US population. The database includes data from all nonfederal, short-term, general, and other specialty hospitals in the US (excluding rehabilitation and long-term acute care hospitals) in the form of de-identified patient information containing demographics, discharge diagnoses, comorbidities, procedures, outcomes, and hospitalization costs.

Participant selection and covariates

We used the International Classification of Diseases, 9th Revision procedure code (ICD-9) of 37.66 (Insertion of Implantable Heart Assist System), in any procedure field among patients 18 years or older. We excluded patients with incomplete data for gender and mortality. To reduce the possibility of data duplication, we excluded patients with an indicator of transfer to another acute-care facility. Patient baseline characteristics included age, sex, race, insurance, and hospital region. Comorbidities and complications were identified using ICD-9 codes in the diagnosis fields and procedures were identified using ICD-9 codes in the procedure fields. Charlson comorbidity index score was categorized as 0, 1, 2 or ≥ 3 [4]. Table 1 lists the ICD-9-CM and Clinical Classification Software codes used to identify comorbidities and procedures.

**Table 1:** ICD-9 codes

Disease	ICD-9 code
LVAD	37.66
Ventricular fibrillation	427.41
Ventricular flutter	427.42
Ventricular tachycardia	427.1
Atrial fibrillation	427.31
AKI	584.5, 584.6, 584.7, 584.8, 584.9
Hypertension	401.1, 401.9, 405.01, 405.09, 401.0, 402.00, 437.2, 642.00, 642.01, 642.02, 642.03, 642.04, 642.10 to 642.24, 642.70 to 642.9
BMI ≥ 25	278.00, 278.02, v85.2, v85.4, 278.01
Mechanical Ventilation	96.04, 96.70, 96.71, 96.72
Hemodialysis	39.95, V45.11, V45.12, V56.0, V56.1
GI bleed	530.7, 530.21, 530.82, 531.0, 531.00, 531.01, 531.2, 531.20, 531.21, 531.4, 531.40, 531.41, 531.6, 531.60, 531.61, 532.0, 532.00, 532.01,532.2, 532.20, 532.21, 532.4, 532.40, 532.41, 532.6, 532.60, 532.61, 533.0, 533.00, 533.01, 533.2, 533.20, 533.21, 533.4, 533.40, 533.41, 533.6, 533.60, 533.61, 534.0, 534.00, 534.01, 534.2, 534.20, 534.21, 534.4, 534.40, 534.41, 534.6, 534.60, 534.61, 535.01, 535.11, 535.21, 535.41, 535.51, 535.61, 535.71, 537.83, 537.84, 578.0, 569.86, 569.85, 562.13, 562.12, 562.03, 562.02, 578.9, 578.1
Arterial embolism and thrombosis	444.01, 444.09, 444.1, 444.21, 444.22, 444.81, 444.89, 444.9
Sepsis and severe Sepsis	995.91, 995.92
Septic Shock	785.52
DIC	286.6

Statistical analysis

A series of comparative analyses were computed to identify trends in patient and hospital characteristics, mortality, comorbidities, and complications. Categorical variables were compared with the chi-square test, and continuous variables were compared with the Wilcoxon rank-sum test. Chi-square test of independence or the Fisher’s exact test was used to assess differences involving categorical variables. One-way analysis of variance tests was used to assess differences between continuous variables. Predictors of in-patient mortality were examined using a hierarchical multivariate binary logistic regression analysis (Table 2). Patient characteristics (i.e., age, sex, and length of stay) were entered in block 1, while comorbidities and complications were entered in block 2. As ≤0.17% of the total sample were diagnosed with a comorbidity of dementia, human immunodeficiency virus/acquired immunodeficiency syndrome (HIV/AIDS), or metastatic solid tumor, each of those variables were omitted from the multivariate analysis. All statistical analyses were performed in R Core Team, 2019, with the Type I error rate set to 0.05 [5].

**Table 2:** Multivariate binary logistic regression model of sepsis and severe sepsis Note. * p < .05, ** p < .001. DM: Diabetes without chronic complications; DMCC: Diabetes with chronic complications; BMI: Body Mass Index; COPD: Chronic obstructive pulmonary disease; SSS: Sepsis and severe sepsis.

Determinant	Sepsis and severe sepsis (0 = No, 1 = Yes)	
	Estimate (SE)	
Block 1		
Age	0.01 (0.01)*	
Sex (reference = male)	-0.04 (0.19)	
Race (reference = white)		
Black	0.22 (0.19)	
Hispanic	0.39 (0.31)	
Asian or Pacific Islander	0.94 (0.54)	
Native American	-0.63 (1.36)	
Other	0.94 (0.30)*	
Length of stay	0.02 (0.00)**	
Model X^2^ (df)	310.74 (8)**	
Nagelkerke R^2^	.24	
Block 2		
Atrial Fibrillation	-0.28 (0.17)	
Hypertension	-0.43 (0.24)	
Acute kidney injury	1.01 (0.20)**	
Ventricular arrhythmia	-0.01 (0.16)	
Hemodialysis	0.33 (0.24)	
Mechanical ventilation	0.85 (0.16)**	
Venous thromboembolism	0.57 (0.23)*	
Gastrointestinal bleed	0.57 (0.23)*	
DM	-0.40 (0.20)	
DMCC	-0.78 (0.45)	
BMI ≥ 25	-0.26 (0.27)	
COPD	-0.59 (0.17)**	
Acute myocardial infarction	-0.61 (0.20)*	
Cerebrovascular disease	-0.04 (0.26)	
Mild liver disease	0.78 (0.17)**	
Hemiplegia or paraplegia	0.11 (0.55)	
Moderate or severe liver disease	0.51 (0.39)	
Model X^2^ (df)	545.53 (25)**	
Nagelkerke ΔR^2^	.41**	

## Results

Sample characteristics, descriptive statistics, and a comparison of trends from 2010 to 2014 are reported in Table 3. Our analysis resulted in 2,359 unweighted records, representing 20% of the population. The total number of LVADs increased steadily from 380 in 2010 to 548 in 2014. The mean age of the sample patients was 55 ± 13.7 years, men (76.8%) and white (59.3%). The highest proportion of patients were in South region (41.4%) and covered by Medicare (45.9%). Over the five-year period, the mean length of stay was 33.18 ± 26.23 days, and the mean total charges for hospitalization was $772,175.90 ± 498,689.70. Although mean length of stay did not vary over the five-year period (p = .250), there was a significant increase in mean hospital charges (p < .001). Overall mortality was 17.1% and did not differ significantly from the year 2010 to 2014 (p = .653). SSS was associated with an increased likelihood of mortality, X^2^ (1) = 387.41, p < .001, OR = 11.09, 95% CI [8.44, 14.62]. The mean length of stay among LVAD patients with SSS was higher (M = 56.20) compared to those without SSS (M = 30.13), t (2355) = 16.38, p < .001. The mean total charges among LVAD patients with SSS was higher (M = 1,209,837.50) compared to those without SSS (M = 716,650.10), t (2334) = 15.90, p < .001.

**Table 3:** Characteristics of left ventricular assist device recipients for index admission by year ^a^One-way ANOVA, ^b^Chi-square test of independence, ^c^Fisher’s exact test. LVAD: Left ventricular assist device; DM: Diabetes without chronic complications; DMCC: Diabetes with chronic complications; BMI: Body mass index; COPD: Chronic obstructive pulmonary disease; DIC: Disseminated intravascular coagulation; SSS: Sepsis and severe sepsis; AET: Arterial embolism and thrombosis.

Variable	Year					p-value	Total
	2010	2011	2012	2013	2014		
Total LVADs	380	426	482	523	548		2359
SSS	36 (9.47%)	55 (12.91%)	57 (11.83%)	65 (12.43%)	65 (11.86%)	.612^b^	278 (11.78%)
Mortality	61 (16.05%)	83 (19.48%)	83 (17.22%)	88 (16.83%)	88 (16.06%)	.653^b^	403 (17.08%)
Age (years), M ± SD	53.69 ± 13.64	55.31 ± 14.11	55.18 ± 13.69	54.41 ± 13.92	56.38 ± 13.25	.037^a^	55.07 ± 13.73
18-34	49 (12.89%)	45 (10.56%)	48 (9.96%)	58 (11.09%)	45 (8.21%)		245 (10.39%)
35-49	70 (18.42%)	80 (18.78%)	99 (20.54%)	112 (21.41%)	99 (18.07%)		460 (19.50%)
50-64	176 (46.32%)	177 (41.55%)	203 (42.12%)	215 (41.11%)	241 (43.98%)		1012 (42.90%)
≥ 65	85 (22.37%)	124 (29.11%)	132 (27.39%)	138 (26.39%)	163 (29.74%)		642 (27.21%)
Male	291 (76.58%)	321 (75.35%)	372 (77.18%)	414 (79.16%)	414 (75.55%)	.615^b^	1812 (76.81%)
Race						.082^c^	
White	240 (63.16%)	268 (62.91%)	285 (59.13%)	291 (55.64%)	315 (57.48%)		1399 (59.30%)
Black	77 (20.26%)	77 (18.08%)	103 (21.37%)	122 (23.33%)	132 (24.09%)		511 (21.66%)
Hispanic	31 (8.16%)	29 (6.81%)	23 (4.77%)	30 (5.74%)	32 (5.84%)		145 (6.15%)
Asian or Pacific Islander	6 (1.58%)	7 (1.64%)	9 (1.87%)	5 (0.96%)	4 (0.73%)		31 (1.31%)
Native American	1 (0.26%)	3 (0.70%)	2 (0.41%)	1 (0.19%)	3 (0.55%)		10 (0.42%)
Other	14 (3.68%)	12 (2.82%)	29 (6.02%)	29 (5.54%)	15 (2.74%)		99 (4.19%)
Unknown	11 (2.89%)	30 (7.04%)	31 (6.43%)	45 (8.60%)	47 (8.58%)		164 (6.95%)
Insurance						.003^c^	
Medicare	157 (41.32%)	199 (46.71%)	224 (46.47%)	236 (45.12%)	267 (48.72%)		1083 (45.91%)
Medicaid	41 (10.79%)	55 (12.91%)	62 (12.86%)	68 (13.00%)	64 (11.68%)		290 (12.29%)
Private	170 (44.74%)	144 (33.80%)	178 (36.93%)	195 (37.28%)	195 (35.58%)		882 (37.39%)
Uninsured	7 (1.84%)	5 (1.17%)	5 (1.04%)	7 (1.34%)	5 (0.91%)		29 (1.23%)
Other	5 (1.32%)	20 (4.69%)	8 (1.66%)	10 (1.91%)	10 (1.82%)		53 (2.25%)
Unknown	0 (0.00%)	3 (0.70%)	5 (1.04%)	7 (1.34%)	7 (1.28%)		22 (0.93%)
Hospital region						.289^b^	
Northeast	70 (18.42%)	105 (24.65%)	82 (17.01%)	101 (19.31%)	103 (18.80%)		461 (19.54%)
Midwest	89 (23.42%)	104 (24.41%)	124 (25.73%)	119 (22.75%)	138 (25.18%)		574 (24.33%)
South	156 (41.05%)	158 (37.09%)	203 (42.12%)	225 (43.02%)	235 (42.88%)		977 (41.42%)
West	65 (17.11%)	59 (13.85%)	73 (15.15%)	78 (14.91%)	72 (13.14%)		347 (14.71%)
Length of stay (days), M ± SD	30.93 ± 24.75	35.22 ± 27.58	33.23 ± 25.85	33.18 ± 24.59	33.12 ± 27.91	.250^a^	33.18 ± 26.23
Total charges (dollars), M ± SD	637,205.27 ± 274,142.86	746,387 ± 465,239.90	791,223.50 ± 531,433.50	820,904.50 ± 546,710.30	819,105.40 ± 544,305.30	< .001^a^	772,175.90 ± 498,689.70
Comorbidities							
Atrial Fibrillation	132 (34.74%)	164 (38.50%)	183 (37.97%)	209 (39.96%)	244 (44.53%)	.039^b^	932 (39.51%)
Hypertension	72 (18.95%)	73 (17.14%)	88 (18.26%)	116 (22.18%)	113 (20.62%)	.301^b^	462 (19.58%)
Acute kidney injury	173 (45.53%)	236 (55.40%)	245 (50.83%)	300 (57.36%)	330 (60.22%)	< .001^b^	1284 (54.43%)
Ventricular arrhythmia	159 (41.84%)	193 (45.31%)	219 (45.44%)	234 (44.74%)	258 (47.08%)	.635^b^	1063 (45.06%)
Hemodialysis	27 (7.11%)	35 (8.22%)	33 (6.85%)	29 (5.54%)	39 (7.12%)	.608^b^	163 (6.91%)
Mechanical ventilation	123 (32.37%)	143 (33.57%)	142 (29.46%)	132 (25.24%)	158 (28.83%)	.049^b^	698 (29.59%)
Aortic stenosis	26 (6.84%)	22 (5.16%)	31 (6.43%)	31 (5.93%)	29 (5.29%)	.802^b^	139 (5.89%)
Venous thromboembolism	26 (6.84%)	37 (8.69%)	43 (8.92%)	42 (8.03%)	40 (7.30%)	.756^b^	188 (7.97%)
Gastrointestinal bleed	24 (6.32%)	35 (8.22%)	40 (8.30%)	38 (7.27%)	39 (7.12%)	.793^b^	176 (7.46%)
Peripheral vascular disease	19 (5.00%)	26 (6.10%)	45 (9.34%)	37 (7.07%)	38 (6.93%)	.141^b^	165 (6.99%)
DM	89 (23.42%)	101 (23.71%)	141 (29.25%)	148 (28.30%)	157 (28.65%)	.123^b^	636 (26.96%)
DMCC	17 (4.47%)	15 (3.52%)	26 (5.39%)	32 (6.12%)	28 (5.11%)	.444^b^	118 (5.00%)
BMI ≥ 25	44 (11.58%)	56 (13.15%)	38 (7.88%)	87 (16.63%)	95 (17.34%)	< .001^b^	320 (13.57%)
COPD	182 (48.42%)	197 (46.24%)	235 (48.76%)	254 (48.57%)	278 (50.73%)	.745^b^	1148 (48.66%)
Acute myocardial infarction	110 (28.95%)	109 (25.59%)	129 (26.76%)	128 (24.47%)	149 (27.19%)	.626^b^	625 (26.49%)
Cerebrovascular disease	39 (10.26%)	31 (7.28%)	25 (5.19%)	49 (9.37%)	55 (10.04%)	.023^b^	199 (8.44%)
Dementia	0 (0.00%)	0 (0.00%)	0 (0.00%)	0 (0.00%)	1 (0.18%)	1.000^c^	1 (0.04%)
Rheumatoid disease	5 (1.32%)	6 (1.41%)	4 (0.83%)	8 (1.53%)	3 (0.55%)	.475^c^	26 (1.10%)
Mild liver disease	47 (12.37%)	81 (19.01%)	102 (21.16%)	89 (17.02%)	101 (18.43%)	.016^b^	420 (17.80%)
Hemiplegia or paraplegia	4 (1.05%)	5 (1.17%)	5 (1.04%)	4 (0.76%)	15 (2.74%)	.089^c^	33 (1.40%)
Renal disease	147 (38.68%)	170 (39.91%)	195 (40.46%)	206 (39.39%)	250 (45.62%)	.164^b^	968 (41.03%)
Moderate or severe liver disease	8 (2.11%)	8 (1.88%)	13 (2.70%)	11 (2.10%)	8 (1.46%)	.728^b^	48 (2.03%)
Metastatic solid tumor	2 (0.53%)	1 (0.23%)	0 (0.00%)	0 (0.00%)	1 (0.18%)	.259^c^	4 (0.17%)
Cancer	11 (2.89%)	9 (2.11%)	13 (2.70%)	13 (2.49%)	10 (1.82%)	.822^b^	56 (2.37%)
HIV/AIDS	1 (0.26%)	0 (0.00%)	2 (0.41%)	0 (0.00%)	0 (0.00%)	.163^c^	3 (0.13%)
Peptic ulcer disease	3 (0.79%)	5 (1.17%)	7 (1.45%)	4 (0.76%)	9 (1.64%)	.653^c^	28 (1.19%)
Charlson score, M ± SD	2.78 ± 1.19	2.77 ± 1.10	2.93 ± 1.24	2.86 ± 1.18	3.01 ± 1.17	.010^a^	2.88 ± 1.18
0	1 (0.26%)	0 (0.00%)	0 (0.00%)	2 (0.38%)	0 (0.00%)		3 (0.13%)
1	55 (14.47%)	51 (11.97%)	55 (11.41%)	63 (12.05%)	49 (8.94%)		273 (11.57%)
2	106 (27.89%)	135 (31.69%)	134 (27.80%)	142 (27.15%)	145 (26.46%)		662 (28.06%)
≥ 3	218 (57.37%)	240 (56.34%)	293 (60.79%)	316 (60.42%)	354 (64.60%)		1421 (60.24%)

An increase in co-morbidities such as atrial fibrillation, acute kidney injury, mechanical ventilation, body mass index ≥ 25, cerebrovascular disease, and mild liver disease was evidenced over the five-year period (all p-values ≤ .049). During the five-year study period from 2010 to 2014 the average incidence of SSS was 11.8%. There was no significant change in the trend of SSS over five years (p = .612). While controlling for age, sex, and LOS, hierarchical multivariate logistic regression revealed significant predictors of SSS were acute kidney injury [Adjusted odd’s ratio (AOR) = 2.75, 95% CI = 1.87, 4.14], mechanical ventilation (AOR = 2.34, 95% CI = 1.70, 3.23), venous thromboembolism (AOR = 1.76, 95% CI = 1.12, 2.75), gastrointestinal bleed (AOR = 1.77, 95% CI = 1.12, 2.76), chronic obstructive pulmonary disease (AOR = 0.55, 95% CI = 0.40, 0.77), acute myocardial infarction (AOR = 0.54, 95% CI = 0.36, 0.80), mild liver disease (AOR = 2.18, 95% CI = 1.55, 3.06).

## Discussion

Infections are a common cause of morbidity and the second most common cause of death in patients with LVAD. It is also one of the leading causes of readmission in these patients [6]. With an increasing use of LVAD devices in patients with heart failure, the study explored the incidence of sepsis and its clinical implications. The findings of this study indicated that the rate of sepsis remained constant over the past few years. With great technological advancements in medicine, improved perioperative care, better understanding of the devices and preventative measures, we would expect the rate of infectious complications to go down. However, despite these advances, the rates have remained the same. Patients who undergo LVAD implantation for destination therapy are more likely to have infectious complications as they tend to be sicker compared to patients who undergo implantation as a bridge to transplant [7]. There were many studies which demonstrated the rates of these infectious complications. In the HeartMate II bridge to transplant trial, a significant proportion of patients had non-LVAD infections (28%), sepsis (20%), and LVAD driveline infections (14%) after LVAD implantation [8]. The HeartMate II destination therapy trial has also demonstrated higher rates of infections, including local non-LVAD infections (49%), sepsis (36%), and LVAD-related infections (35%) in the treatment arm [2]. Data from INTERMACS registry shows that pneumonia and sepsis are the most common infections implicated in patients with LVAD, followed by driveline infections that is mostly a late onset infection [9]. The risk of developing a blood stream infection followed by sepsis is highest in the perioperative period.

Using the Charlson co-morbidity index to compare the comorbidities in patients from 2010 to 2014, there was evidence showing increasing trends of co-morbid conditions over the years [4]. Patients had significantly higher rates of atrial fibrillation, acute kidney injury, mechanical ventilation, BMI >25, mild liver disease and cerebrovascular disease. Increasing trends of these conditions demonstrate that the baseline health of patients is poorer, which could explain the lack of improvement in the rate of infectious complications. Hence the criteria which deem eligibility for mechanical circulatory support should be revised, being mindful of the burden of these disease among patients to improve outcomes. Adequate management of individual co-morbid conditions and appropriate patient optimization can be beneficial in achieving desirable outcomes. Multivariate analysis demonstrated that determinants like acute kidney injury, mechanical ventilation, venous thromboembolism, gastrointestinal bleeding, mild liver disease play a role in causation of sepsis. However, a few studies have demonstrated that the presence of renal dysfunction, age, sex was not a significant risk factor in the development of infection after LVAD implantation. Interestingly, it was also seen that patients with any type of post-implantation infection had a trend towards higher BMI compared with the patients who did not have an infection [10]. There was a study that demonstrated that women had fewer infections compared to men among patients using LVAD as a bridge to transplantation, for reasons unknown [11]. A study by Simon et al. demonstrated no significant host factor responsible for developing LVAD associated infectious complications, however a subgroup analysis revealed that patients with LVAD-related bloodstream infection were more likely to have diabetes mellitus compared with all other LVAD recipients and this was attributed to microvascular insufficiency and neutrophil dysfunction [12].

The microbiological profile of LVAD-related infections revealed predominance of Staphylococcal and pseudomonal species in patients with continuous flow devices. There were studies previously that demonstrated these pathogens as a common cause of driveline infections [13, 14]. These pathogens produce biofilm that is highly resistant to both innate and adaptive host defense mechanisms by forming a protective barrier around their colonies [15]. As mentioned previously, production of biofilm makes it challenging to eradicate the infection. The risk of developing the bloodstream infection and sepsis is highest in the perioperative period, while percutaneous driveline infection is a late onset infection. It was seen that pneumonia and sepsis are the most common infectious complications in patients on continuous flow-LVAD (23% and 20%, respectively), followed by percutaneous site infections, which occurs in approximately 19% of the recipients by one year post-implant, and is associated with an increased risk of mortality [16]. Analysis of infectious complications in the REMATCH trial revealed that the risk of sepsis peaked early (within three weeks) after implantation [17].

Our study demonstrated that SSS was associated with an increased in-hospital mortality, greater length of stay (LOS), and higher hospital-related charges compared to those without SSS. This could be multifactorial in the setting of sepsis and also underlying comorbidities that would increase the mortality by itself. There were a few studies that showed no significant long-term outcomes in people with these infectious complications, although they showed increased length of hospital stay [12, 18-19]. A few studies have shown that LVAD infections have significant association with poor survival after adjusting for age and co-morbidities [20]. LVAD infections were reported as a leading cause of hospital readmission in some [6, 10, 21-23]. Data from the Eighth INTERMACS registry demonstrated no change in the survival curve since 2013 [9]. Therefore, early detection and treatment of localized infections may prevent development of sepsis and improve outcomes and survival in the post-transplant phase. It was shown that although survival to transplantation was diminished in LVAD patients with sepsis, if successfully transplanted, the post-transplant survival rate was unaffected [24, 25]. A study by Simon et al. revealed that although LVAD-related infections delayed transplantation but they did not prevent it. It is important to note that the long-term outcomes remained the same in patients with or without infectious complications [12]. Various preventive measures have been proposed to reduce the infectious complications of LVAD such as use of perioperative antibiotics, minimizing the tension at the exit site by use of anchoring devices and appropriate surgical site care using chlorhexidine. Use of chronic prophylactic antibiotics such as doxycycline or levofloxacin is not proven to be beneficial in decreasing the incidence of driveline infections compared to usual care [26]. Surgical techniques such as tunneling of driveline into the fascia of musculus rectus abdominus, and double tunneling could potentially reduce the incidence of driveline infections [18, 27].

There are no specific guidelines for treatment of these infections. Treatment largely depends on bacterial cultures from the wound, blood and their respective sensitivities. Mild infections can be managed by increasing frequency of dressing changes, local wound care and close monitoring. If the patient has signs of systemic infections, management should be more targeted and guided by sensitivity-based antimicrobial therapy, imaging tests and surgical intervention such as debridement and re-tunneling of the device. Chronic suppressive antibiotics are often used in patients with recurrent VAD specific infections, but benefit remains ambivalent as approximately one-third of the patients have recurrence despite antibiotics [28]. Device exchange can be performed for severe cases; however, recurrence of infection remains common with this as well [25, 29]. Although better clinical outcomes are being achieved with continuous flow devices, sepsis/severe sepsis still remains a major complication in patients with LVAD implantation. Better patient selection, revision of eligibility criteria based on the other co-morbid conditions, appropriate management of patient comorbidities and strict adherence to infection control guidelines should be ensured to improve patient outcomes.

Despite including a large sample size NIS database has its own set of limitations. Observations pertain to the population on LVADs in the United States and may not be generalizable to populations in other countries. Sepsis and severe sepsis were identified from ICD-9 codes in diagnoses fields. As this study was based upon a database, we could not differentiate pulsatile flow versus continuous flow-LVADs. However, considering the FDA pattern and utilization rates, we presumed most of them received CF devices from 2010 onwards [27]. There is a lack of information regarding the timing and severity of infections, co-morbidities, pre-operative risk, medications, blood culture results, echocardiogram data, laboratory data, and endoscopy results. The database also does not contain post‐discharge data on long‐term outcomes. Lastly, this cohort was defined using diagnosis codes and may be subject to misclassification, but this is unavoidable in an administrative database analysis.

## Conclusions

These results show that, even though the rate of incidence of sepsis has remained constant, it remains a common finding. In addition, SSS is associated with worse clinical outcomes. We have identified seven clinical conditions associated with SSS. This information could provide clues to identify high-risk groups and potentially improve patient selection or argue for earlier detection in certain clinical scenarios. Further trials looking at the surgical techniques, peri-operative and post-operative care and better patient selection can help reduce the infectious complications and improve clinical outcomes.
